# Improved quantification of T2* relaxation in magnetic resonance imaging

**DOI:** 10.1186/1532-429X-13-S1-P47

**Published:** 2011-02-02

**Authors:** Einar Heiberg, Jane Sjögren, Håkan Arheden

**Affiliations:** 1 Department of Clincial Physiology, Lund, Sweden

## Introduction

MRI can acquire images from which myocardial tissue properties can be derived. One such parameter is T2* relaxation. Currently there is software available that calculate T2* values and generate tissue maps. However, they do not give information of certainty and they have problems with numerical stability for low T2* values.

## Purpose

The purpose of this study was therefore to develop a freely available software for quantification of T2* relaxation that also generates certainty estimates.

## Methods

The new method has been validated in digital phantoms with actual echo times and noise levels taken from 5 patients. To achieve numerical stability at low T2* values, a novel hybrid curve fitting method was developed. For low values the residuals were weighted with the signal intensity and for high values standard exponential fitting was used. The transition between high and low regions was found by looking at the ratio between the mean and standard deviation of samples with TE >20 ms. In a pure Rician noise distribution this ratio is 1.3, ratios below this were therefore taken as indication of low T2* values. A curve fitting error map that shows the mean percentage curve fitting error for each sample was also calculated. The algorithm was implemented in the freely available cardiac image analysis software Segment (http://segment.heiberg.se). The method was validated on computer phantoms (n=140 000) with different noise levels. True T2* values were in the range of 1-100 ms, 12 echoes with TE ranges of 1-44 ms. Statistics were derived for the relative error in the T2* quantification as a function of the curve fitting error.

## Results

See figures 1,2,3.

**Figure 1:**
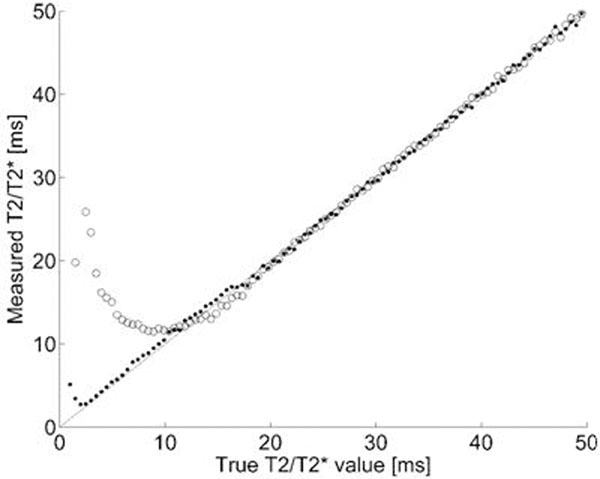
Correlation plot for the new hybrid method in (n=100) computer phantoms. Open circles are standard curve fitting and filled circles are the new hybrid method. The new hybrid method outperforms standard fitting for T2* values <15 ms.

**Figure 2:**
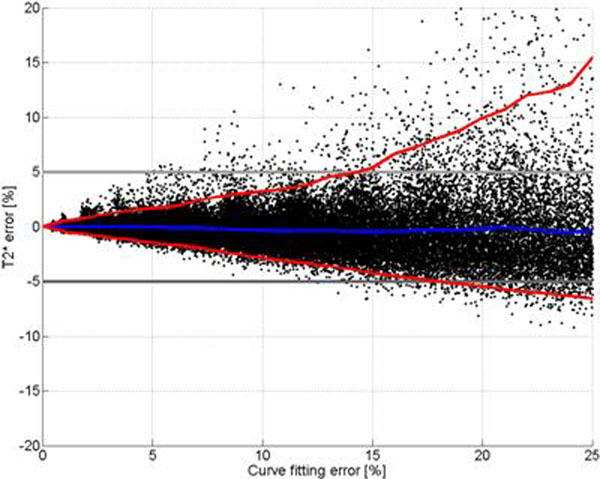
Relative T2* quantification error as a function of curve fitting error. Red lines shows 95% confidence interval. Blue line shows mean error. T2* values can with 95% confidence be quantified with a relative error of <5% if the fitting error is less than 15%.

**Figure 3:**
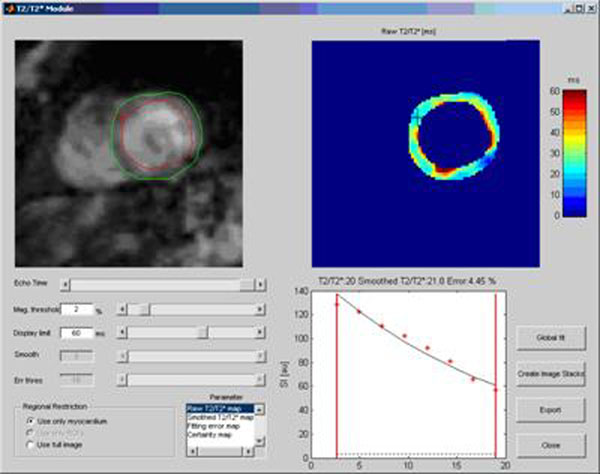
Example of the user interface.

## Conclusions

We have developed a method that quantifies T2* with improved accuracy of low T2* values and that also provides accuracy information. T2* values can be quantified with a relative error of <5% if the curve fitting error is less than 15%.

